# Supplementary material for: RNA-Seq and 16S rRNA Reveals That Tian–Dong–Tang–Gan Powder Alleviates Environmental Stress-Induced Decline in Immune and Antioxidant Function and Gut Microbiota Dysbiosis in Litopenaeus vannami
Source: Antioxidants (Basel). 2023 Jun 12;12(6):1262. doi: 10.3390/antiox12061262 (PMC10295192; doi:10.3390/antiox12061262)
Supplement: Supplementary file 1 [file antioxidants-12-01262-s001.zip › antioxidants-2409685-supplementary materials.pdf]

Supplementary materials

RNA-seq and 16S rRNA reveal that Tian−Dong−Tang−Gan Powder alleviates ammonia stress and nitrite stress-induced decline in immune and antioxidant function and gut microbiota dysbiosis in *Litopenaeus vannami*

Xiao−Dong Xie [1], Ying Zhou [1], Yu−Bo Sun [1], Shou−Li Yi [1], Yi Zhao [1], Qi Chen [1], Ying−Hong Xie [1], Mi−Xia Cao [2], Mei−Ling Yu [1], Ying−Yi Wei [1], Ling Zhang [3,*] and Ting−Jun Hu [1,*]

[1] College of Animal Science and Technology, Guangxi University, Nanning 530005, China; 1718304007@st.gxu.edu.cn (X.−D.X.); zhouying@st.gxu.edu.cn (Y.Z.); yubosun@st.gxu.edu.cn (Y.−B.S.); yishouli@st.gxu.edu.cn (S.−L.Y.); zhaoyi@st.gxu.edu.cn (Y.Z.); chenqi@st.gxu.edu.cn (Q.C.); ying-hongxie@st.gxu.edu.cn (Y.−H.X.); yumeiling@gxu.edu.cn (M.−L. Y.); weiyingyi@gxu.edu.cn (Y.−Y.W.)

[2] College of Animal Science, Anhui Science and Technology University, Chuzhou 233100, China; caomixia66668888@st.gxu.edu.cn

[3] Guangxi Scientific Research Center of Traditional Chinese Medicine, Guangxi University of Chinese Medicine, Nanning 530200, China

* Correspondence: zhangl2011@gxtcmu.edu.cn (L.Z.); tingjunhu@gxu.edu.cn (T.−J.H.); Tel.: +86-771-3235635 (L.Z.); +86-771-3235635 (T.H.); Fax: +86-771-3270149 (L.Z.); +86-771-3270149 (T.−J.H.)

Table S1 Primers used for gene expression analyses by qRT-PCR.

Name	Sequence (5′-3′)	Amplicon size (bp)	Source
β-actin	F- GCCCATCTACGAGGGATA<br>R-GGTGGTCGTGAAGGTGTA	121	XR_003476362.1
Sirt1	AATAGAAGATGCCGTTAG<br>TATAGGTTGCGGTGGT	97	XM_027362423.1
fdxr	AAGAAGCATAGGCTACAAG<br>TCCATCCGCTGACATAAA	125	XM_027360895.1
HO	CGGATACAAGCCAAGGG<br>GCCGCCAGATAAGAGCC	124	XM_027376281.1
hsp90b1	TGTTGGCTTCTACTCG<br>CCTCGCTTTAGGGTGT	144	XM_027377823.1
Pu	CCACCTGGTTCCCTTCTG<br>TTCAACAATCCTGGCTATCTT	91	XM_027357632.1
GOT1	TGCTGAGCGTGGATTT<br>AGGAGCCTCGGACAAG	155	XM_027383673.1
VEGF	AATGAACGCAAGAAACTCG<br>CACAGCATCCACCGCACT	151	XM_027355751.1

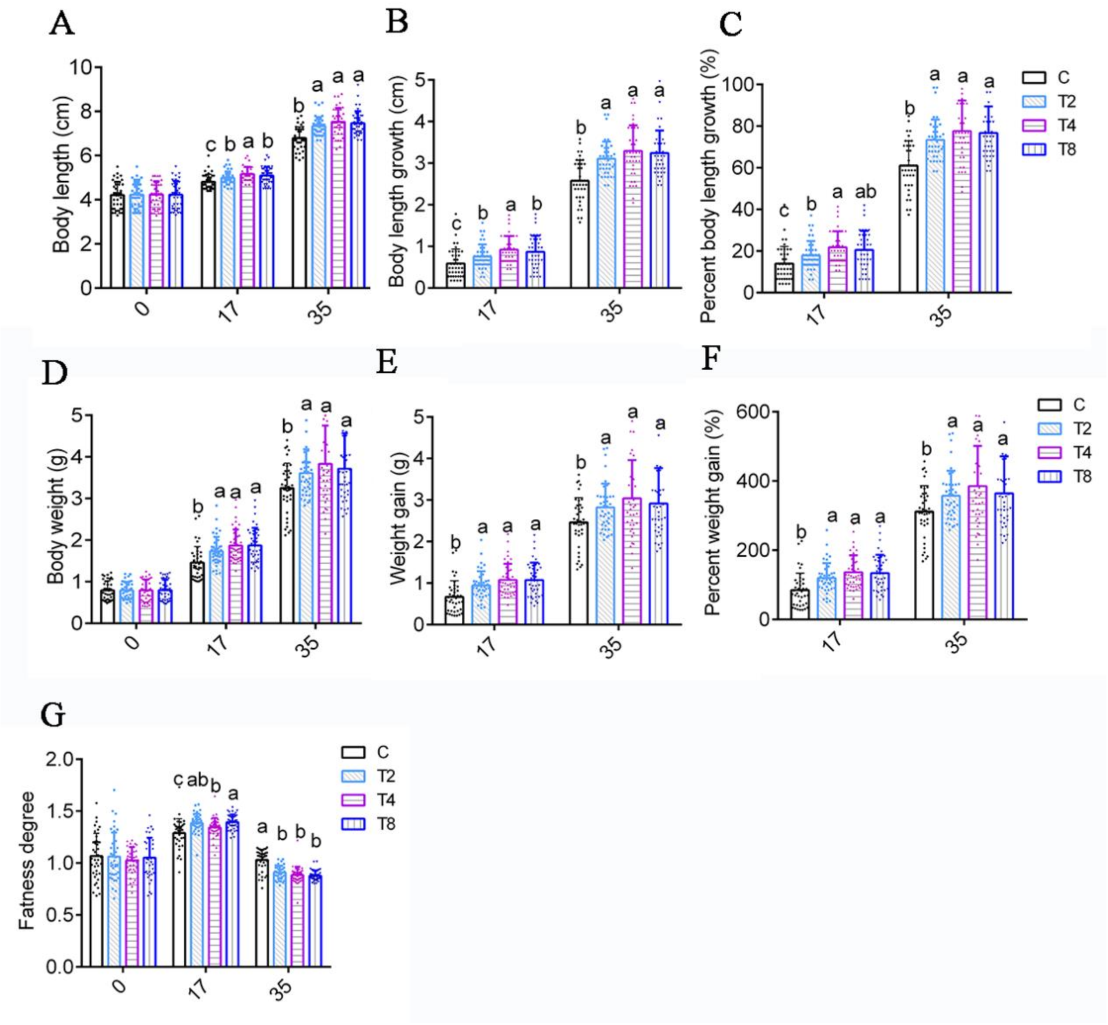

Fig. S1. The body index changes of the *L. vannamei* in the feeding experiment. Note: the parameters were calculated as follows: Weight gain (WG; g) = Wt – W0; Percent weight gain (PWG; %) =100 × (Wt – W0)/W0; Body length growth (BLG; cm) = Lt –L0; Percent body length growth (PBLG; %) =100 × (Lt – L0)/L0; Fatness degree (FD) = 100 × Wt/Lt3; Where Wt is the final body weight (g), W0 is the initial body weight (g), Lt is the final body length (cm), L0 is the initial body length, and t is the experimental duration in days.

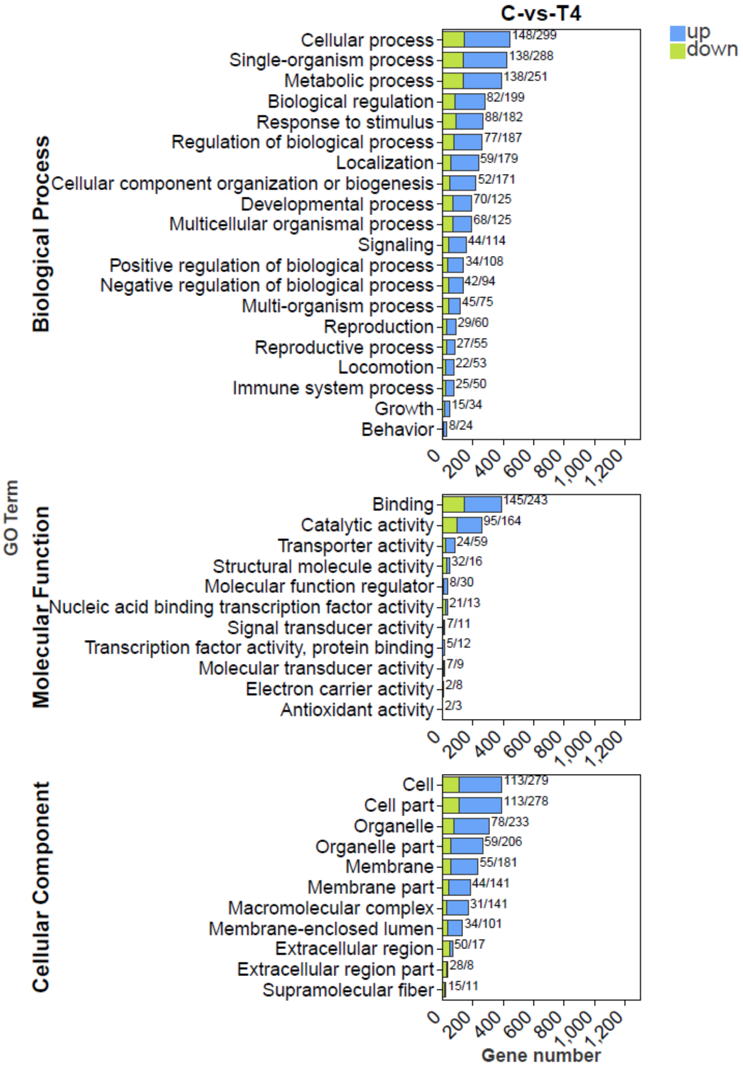

Figure S2. The GO enrichment analysis results of differential genes in TDTGP-4 and blank control groups. Notes: C: blank control group; T4: TDTGP-4 group.

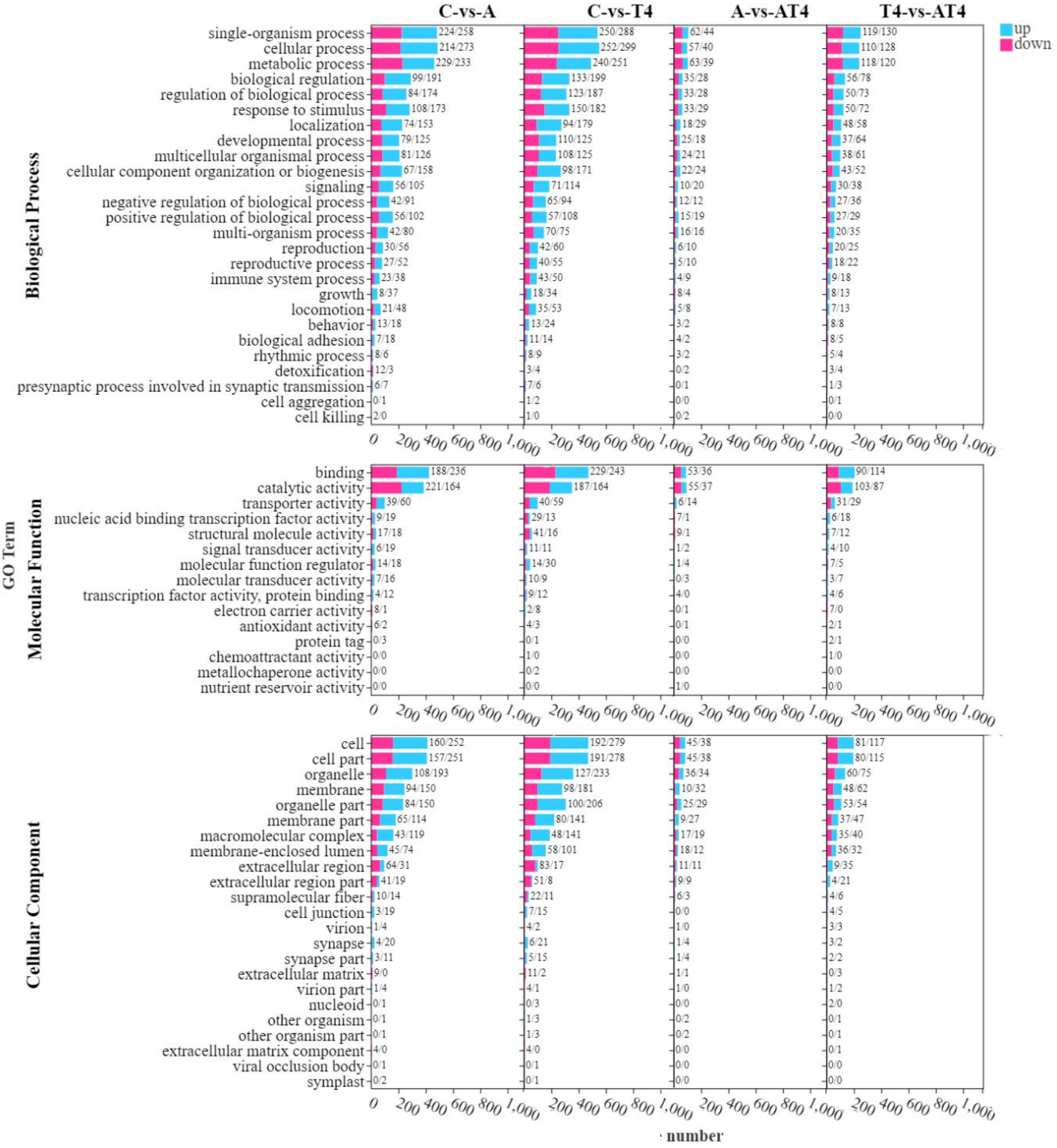

Figure S3. The GO enrichment analysis results of differential genes in the ammonia stress test.

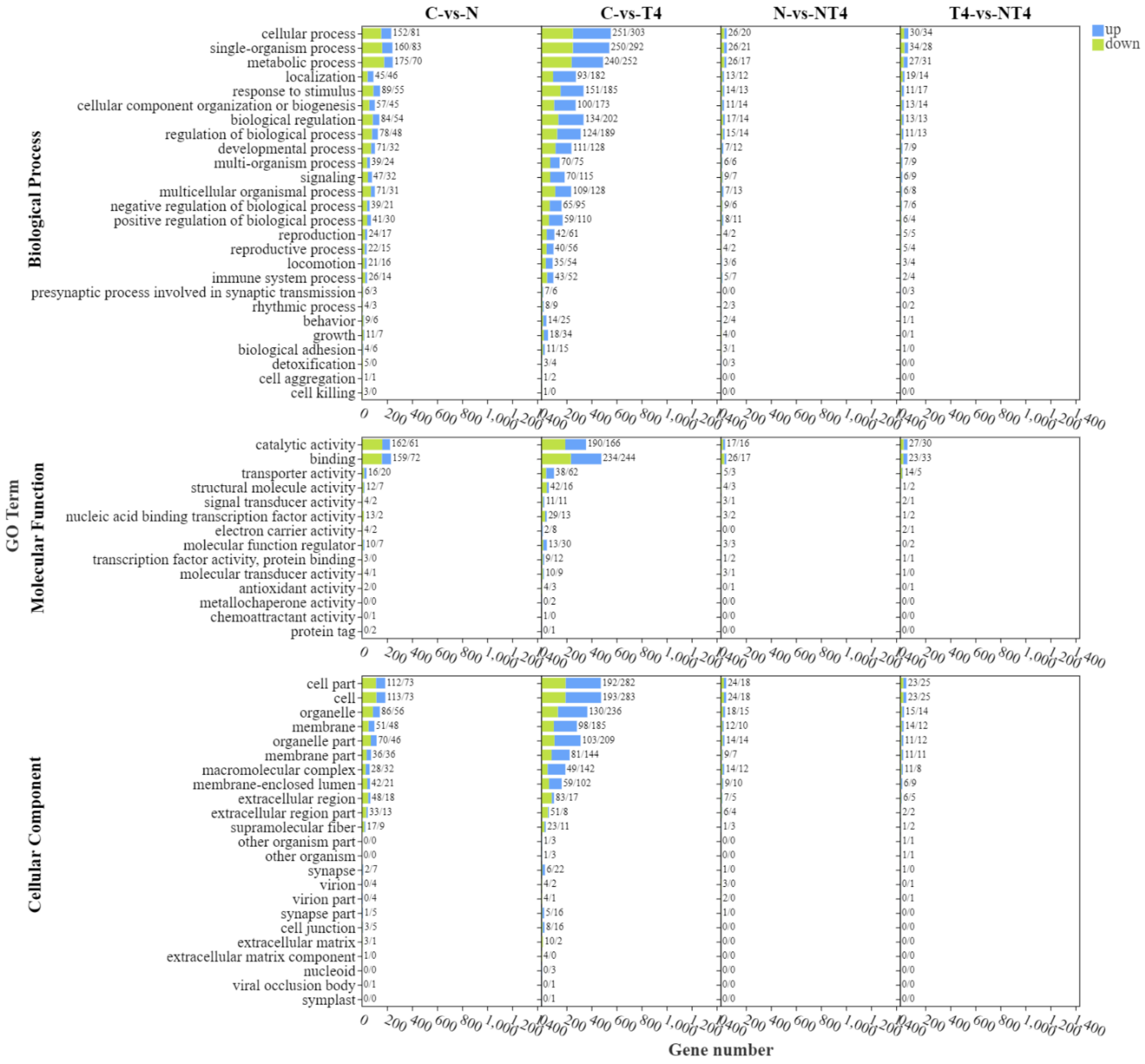

Figure S4. The GO enrichment analysis results of different genes in nitrite stress test.

Analysis of bacteriostatic effect of TDTGP on *Vibrio* in vitro

1. Methods

Oxford cup bacteriostatic test and constant broth dilution test were used to detect the bacteriostatic zone and minimum inhibitory concentration (MIC) of the TDTGP on Vibrio. The MIC of the TDTGP was determined. Simply, using aseptic inoculation needle to pick up a small amount of the nitrite stress group shrimp feces to facilitate the isolation of Vibrio on thiosulfate citrate bile salts sucrose agar (TCBS) medium, after 24 hours of culture at 37 ℃, appropriate amount of single colony was selected and inoculated in 5 mL aseptic LB broth medium to expand 12 h~16 h to 1 × 10^5 pathogens (OD 600 nm=0.2). 2 g TDTGP was dissolved in 20 mL 0.9% sodium chloride solution, then was filtered and sterilized by 0.22 um filter head to prepare the original solution. 2 mL of aseptic LB broth was taken, and the TDTGP solution was diluted by double dilution method, and the final concentration was 50~0.049 mg·mL^{-1}. 20 μL of Vibrio solution containing 1 × 10^5 vibrio was added to diluted LB broth containing the TDTGP. After being cultured at 37 ℃ for 20 h, the absorbance value (600 nm) was determined by enzyme labeling instrument, and the MIC was obtained. At the same time, the aseptic cotton swab was dipped in 1 × 105 vibrio solution (OD 600 nm=0.2) and evenly coated on TCBS medium, and put into 4 sterile oxford cups (inner diameter 6 nm, outer diameter 8 nm, high 10 nm round tubule), and 200 μL of the TDTGP solution of 100, 50, 25 and 12.5 mg·mL^{-1} were added and cultured at 37 ℃ for 24 h, and the bacteriostatic zone was determined.

2. Results

The results of the oxford cup bacteriostatic test showed that no bacteriostatics zone was observed in the range of 100~12.5 mg·mL^{-1} concentration (Fig. 5E). The results of constant broth dilution test showed that there was *Vibrio* growth in LB broth medium with TDTGP concentration of 50~0.049 mg·mL^{-1} (Fig. 5F), indicating that TDTGP had no obvious inhibitory effect on *vibrio* growth within 50 mg·mL^{-1}.

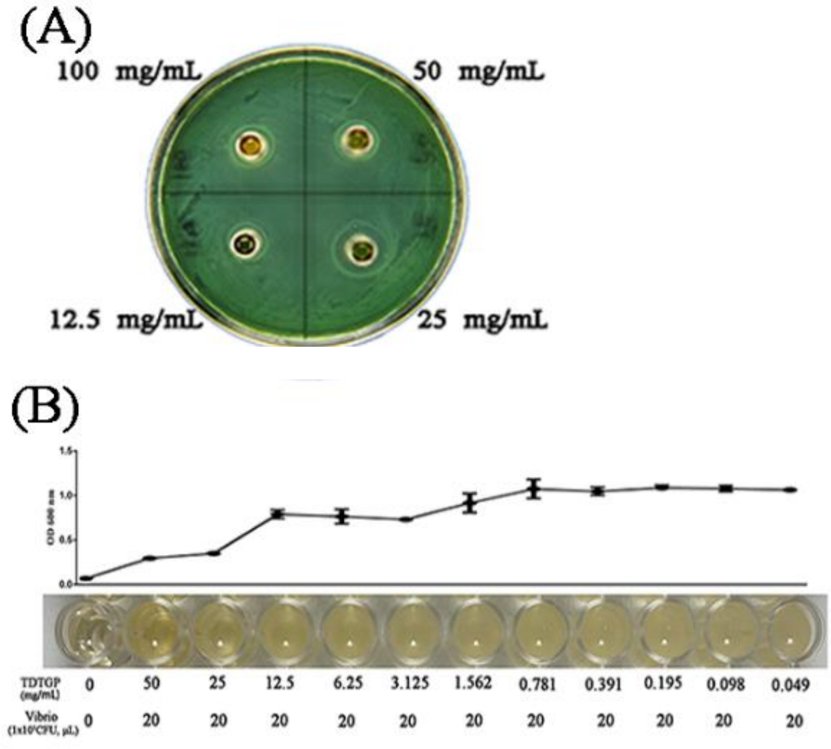

Figure. S5. The results of oxford cup test and constant broth dilution test. Note: (A): Oxford cup test, 100 mg/mL-25 mg/mL is TDTGP 100 mg/mL-25 mg/mL; (B): Constant broth dilution test.
